# Looking back: three key lessons from 20 years of shaping Japanese genome research regulations

**DOI:** 10.1038/s10038-021-00923-z

**Published:** 2021-05-10

**Authors:** Jusaku Minari, Megumu Yokono, Kayo Takashima, Minori Kokado, Ryuichi Ida, Yutaka Hishiyama

**Affiliations:** 1 Uehiro Research Division for iPS Cell Ethics, Center for iPS Cell Research and Application (CiRA), Kyoto University, Kyoto, Japan; 2 School of Social Sciences, Waseda University, Tokyo, Japan; 3 Laboratory of Social Sciences, Kobe Pharmaceutical University, Hyogo, Japan; 4 Shiga University, Shiga, Japan; 5 National Institute of Science and Technology Policy, Tokyo, Japan

**Keywords:** Social sciences, Ethics

Since August 2018, a governmental committee in Japan formed jointly by three relevant ministries has reviewed two existing governmental ethical guidelines for revision: Ethical Guidelines for Human Genome/Gene Analysis Research (Genome Guidelines) and Ethical Guidelines for Medical and Health Research Involving Human Subjects. A single set of guidelines integrating the two, keeping the latter as the main framework, was released in March 2021. The Genome Guidelines, established in 2001 ahead of two other governmental ethical guidelines on epidemiological research (2002) and clinical research (2003), have largely contributed to regulating genome research in Japan. This article, which reviews 20 years of experience regarding the Genome Guidelines, suggests three key lessons for future regulatory debates and practices. Through this article, the authors, who have been closely involved either in elaborating and/or in applying the Genome Guidelines, advocate for inquiring into the true nature of ethical regulation from the perspective of experts in the field of biomedical research ethics.

The first lesson concerns how to manage relationships between fundamental concepts and specific procedures. In 2000, the Bioethics Committee of the Council for Science and Technology established the Fundamental Principles of Research on the Human Genome (Fundamental Principles), thereby clarifying the conceptual ethical framework for the human genome and its related research. The 2001 Genome Guidelines presented concrete practical procedures, including the requirements for informed consent, research protocols and ethical review. Over the last 20 years, these principles and guidelines have been recognised as national norms, but their respective roles have gradually changed. While the fundamental principles have remained unchanged (only becoming less referenced in the practice of genome research), the 2001 Genome Guidelines have been repeatedly and dynamically revised to address scientific, ethical and social issues, becoming more detailed and procedurally concretised. These phenomena could be partly regarded as a formalisation of the principles elaborated upon in the Fundamental Principles and an increase in the procedural formalities (not necessarily linked with the conceptual background) of the 2001 Genome Guidelines. To emphasise the guidelines as practical, ethical and social instruments—not as mere checklists and flow charts—regulation drafters should continuously consider and present the historical background, reasons and implications underlying major stipulations, with connecting fundamental ideas and specific procedures, for their future developments.

The second lesson concerns the specificity of the non-legally binding nature of the guidelines. Until recently, soft laws (i.e., non-legally binding guidelines) have been widely adopted in Japanese medical research [1, 2] to ensure regulatory responsiveness and flexibility regarding scientific advancement and societal changes. The Genome Guidelines have undergone three complete and four partial revisions, two of which were prompted by the enactment (2003) and revision (2015) of the *Act on the Protection of Personal Information* (APPI) [3–5]. Although the APPI was intended to address commercial or business activities, rather than academic research, it has significantly influenced the Genome Guidelines’ provisions regarding the collection, storage and transfer of genetic/personal data. Incorporating the APPI’s provisions into the Genome Guidelines could ensure legal protection. However, it could also cause regulatory instability and confusion for research developments, given that provisions regarding research-related genetic/personal data handling may be irregularly changed due to the APPI’s amendment and related regulations, which are also influenced by the enactments and their revisions in other jurisdictions (e.g., the *European Union General Data Protection Regulation*). Specific hard laws may be necessary to stabilise an appropriate genome research environment while considering international regulatory coordination. Legislation by the Diet members could enable the public to be involved in the creation of regulations. Furthermore, new establishments and frequent revisions of various governmental guidelines and recent legislations in specific fields, such as clinical research and regenerative medicine, could create excessive burdens for the rapid implementation of a particular regulation within the research community and for the governmental administration and management of numerous regulations. To avoid creating a regulation ‘maze’ and to build sustainable regulatory research environments, key stakeholders should consider harmonising the relevant regulations and optimising ethical research developments.

The last lesson concerns the ethical framework of biomedical data research to protect human subjects. While the conventional ethical framework of biomedical research is based on considerations of physical rather than informational harm [6], genome research development, including data sharing and biobanking, has increasingly questioned the privacy, confidentiality and security of genome information in light of research ethics. Conversely, influenced by the 1980 Organisation for Economic Co-operation and Development Guidelines on the Protection of Privacy and Transborder Flows of Personal Data, Japan enacted the APPI to ensure the appropriate use and protection of personal data. Consequently, these two streams of research ethics and personal data protection have been incorporated into the Genome Guidelines, as represented in the coexistence of ‘informed consent’ (concerning research) and ‘consent’ (concerning data protection). Simultaneous duplication of the rules of applying legally binding privacy legislation and observing nonbinding ethical regulations has made it more difficult to adjust research ethics and privacy protection. To solve this issue, at least the ethical and legal norms of ‘data subjects’ should be explored, specifically in the case of research using human-derived data without intervention on human subjects or invasion of privacy (secondary use research) [7, 8]. In addition, the implications of applying traditional ethical principles on human subject research to this type of research should be examined while considering matters regarding family, community and ethnicity, where online approaches, including dynamic consent, may contribute to addressing these challenges [9].

Although some issues remain unresolved, the new guidelines embody several major changes: introducing online consent; clarifying returning research results; and promoting single/central ethics review(s) in multi-centre research. Given the increasing growth of life sciences, the newly integrated guidelines were renamed Ethical Guidelines for Life Science, Medical, and Health Research Involving Human Subjects. Notably, governmental guidelines are shaped with an ‘administrative drafting technique’, so their language, expressions and embodiments are often constrained. Thus, when research communities proactively interpret and apply the new guidelines in a practical sense while carefully considering the situation of research participants, these new guidelines must be useful and effective. Finally, since the protection of human subjects and data is related to the public at large, public trust and engagement initiatives [10] could contribute to further sustainable development in biomedical research fields.

## References

[1] Slingsby BT, Nagao N, Akabayashi A (2004). Administrative legislation in Japan: guidelines on scientific and ethical standards. Camb Q Health Ethics.

[2] Tashiro S (2010). Unintended consequences of “soft” regulations: the social control of human biomedical research in Japan. Int J Jpn Sociol.

[3] Minari J, Chalmers D, Kato K (2014). Return of genetic research results: the Japanese experience and its implications for the international debate. SCRIPTed.

[4] Nagai H (2017). Development of personal data handling policy in human genome research: a historical perspective in Japan. Asian Bioeth Rev.

[5] Yamamoto N, Fujita T, Kawashima M, Wittig J, Suzuki M, Kato K (2018). The inclusion of genomic data in the 2015 revision of Japan’s Protection of Personal Information Act: protection of wider range of genomic data as our next challenge. J Hum Genet.

[6] Kaye J (2011). From single biobanks to international networks: developing e-governance. Hum Genet.

[7] Metcalf J, Crawford K (2016). Where are human subjects in Big Data research? The emerging ethics divide. BDS.

[8] Nicol D, Eckstein L, Bentzen HB, Borry P, Burgess M, Burke W (2019). Consent insufficient for data release. Science.

[9] Teare HJA, Prictor M, Kaye J. Reflections on dynamic consent in biomedical research: the story so far. Eur J Hum Genet. 2020;1–8. 10.1038/s41431-020-00771-z. 10.1038/s41431-020-00771-z PMC7695991 33249421

[10] Hishiyama Y, Minari J, Suganuma N (2019). The survey of public perception and general knowledge of genomic research and medicine in Japan conducted by the Japan Agency for Medical Research and Development. J Hum Genet.

